# Carboplatin therapeutic monitoring in preterm and full-term neonates

**DOI:** 10.1016/j.ejca.2015.07.011

**Published:** 2015-09

**Authors:** Gareth J. Veal, Julie Errington, James Hayden, David Hobin, Dermot Murphy, Rachel M. Dommett, Deborah A. Tweddle, Helen Jenkinson, Susan Picton

**Affiliations:** a Northern Institute for Cancer Research, Newcastle University, Newcastle upon Tyne NE2 4HH, UK; b Alder Hey Children’s NHS Trust, Liverpool L12 2AP, UK; c Birmingham Children’s Hospital, Birmingham B4 6NH, UK; d Royal Hospital for Sick Children, Glasgow G3 8SJ, UK; e Bristol Royal Hospital for Children, Bristol BS2 8BJ, UK; f Great North Children’s Hospital, Newcastle upon Tyne NE1 4LP, UK; g Leeds General Infirmary, Leeds LS1 3EX, UK

**Keywords:** Neonates, Retinoblastoma, Neuroblastoma, Chemotherapy, Carboplatin, Therapeutic drug monitoring

## Abstract

**Introduction:**

Administration of the most appropriate dose of chemotherapy to neonates is particularly challenging and frequently not standardised based on any scientific rationale. We report the clinical utility of carboplatin therapeutic drug monitoring in preterm and full-term neonates within the first month of life.

**Methods:**

Carboplatin therapeutic monitoring was performed to achieve target drug exposures area under the plasma concentration–time curve (AUC values) in nine preterm and full-term neonates diagnosed with retinoblastoma or neuroblastoma treated over an 8 year period. Carboplatin was administered over 3 days with therapeutic drug monitoring utilised to target cumulative AUC values of 5.2–7.8 mg/ml min.

**Results:**

AUC values achieved were within 15% of target values for the individual courses of treatment in all but one patient (12/13 courses of treatment), with dose modifications of up to 215% required to achieve target AUC values, based on initial mg/kg dosing schedules. Carboplatin clearance determined across three consecutive chemotherapy courses in two patients increased from 3.4 to 7.1 ml/min and from 7.2 to 16.5 ml/min, representing increases of 210–230% over several weeks of treatment. Complete remission was observed in 8/9 patients, with no renal toxicity reported and only one patient experiencing ototoxicity.

**Conclusion:**

The study highlights the benefits of utilising therapeutic drug monitoring to achieve target carboplatin AUC values in preterm and full-term neonates treated within the first few weeks of life, particularly in view of marked increases in drug clearance observed over consecutive chemotherapy courses. In the absence of therapeutic drug monitoring, body-weight based dosing is recommended, with dosing guidance provided for both approaches to inform future treatment.

## 1 Introduction

The treatment of cancer in very young children represents a major clinical challenge. Developmental physiological changes within the first few weeks of birth have the potential to markedly impact drug disposition, making selection of the most appropriate dosing regimen particularly difficult [1–4]. While dose reductions are commonplace for the vast majority of anticancer drugs utilised in infants and very young children, with reduced dosing regimens commonly defined as being applicable below a specific age or body weight, these dose reductions are largely defined for children between 3 and 12 months of age [5]. The treatment of preterm and full-term neonates in the first few weeks of life needs to be approached judiciously, with the aim of achieving meaningful drug levels to exhibit anti-tumour efficacy, but without adversely affecting the developing child.

A key challenge in this area is the limited amount of data available concerning anticancer drug disposition in the neonatal patient population. However, for those drugs where studies have been reported, clearance values have frequently been shown to differ markedly from those seen in older children and adults [6]. Indeed, this is not surprising when we consider the continuing development and maturation of renal and hepatic function within the first few weeks of life, which may significantly impact on drug metabolism and elimination [7,8]. The generation of an increased volume of drug disposition data in this patient population is essential if we are to positively impact on the treatment of preterm and full-term neonates with cancer through the provision of meaningful dosing regimens based on ‘real world’ data.

The platinum agent carboplatin represents one of relatively few anticancer drugs where information gathered from clinical pharmacology studies has directly impacted on its clinical use in children with cancer. As removal of carboplatin from the body occurs almost exclusively via elimination of unchanged drug in the urine, initial doses are commonly based on renal function as defined by the glomerular filtration rate (GFR) of the patient [9,10]. In addition, exposure to carboplatin is commonly targeted to a defined area under the plasma concentration–time curve (AUC), which has been shown to more closely correlate with clinical parameters including toxicity and response than the dose administered [11,12].

Based on our current knowledge of the pharmacology of carboplatin, changes in kidney function are highly likely to impact on drug disposition. At birth renal function is anatomically and functionally immature, with marked increases during the first 2 weeks of life due to changes in renal vascular resistance and active nephrogenesis occurring between 9 and 36 weeks from birth, with accompanying changes in renal blood flow [13,14]. GFR values of 2–4 ml/min/1.73 m^2^ are commonly observed in full-term neonates, with values as low as 0.6–0.8 ml/min/1.73 m^2^ in preterm neonates [14,15]. While the kidney of the newborn is sufficient for normal growth and development, it provides limited adjustment to a stressful catabolic state which may be observed in sick preterm infants [16]. These changes with advancing gestational and postnatal age provide real potential for differences in drug clearance to be observed in neonates as compared to older children. In this respect we previously published a case report of a preterm infant treated with carboplatin, highlighting the potential for marked changes in drug clearance with age and requirement for increasing doses of carboplatin to achieve defined target AUC values [17].

In the current study we report on the wider clinical utility of carboplatin therapeutic drug monitoring in nine preterm and full-term neonates being treated for retinoblastoma and low risk neuroblastoma within the first weeks of life. Publication of these data in a small but significant patient population allow us to provide clear guidance for clinicians which will positively impact on the future treatment of preterm and full-term neonates with carboplatin. This is clearly an important issue considering a recent report that younger children are at an increased risk of experiencing ototoxicity following treatment with carboplatin [18].

## 2 Patients and methods

### 2.1 Patient treatment

Nine preterm and full-term neonates, studied over an 8 year period, received carboplatin treatment at six clinical centres as part of their standard treatment. Patients were aged between 3 days and 3 weeks old (for full-term neonates) or gestational ages of 52 weeks (born at 28 weeks), 35 weeks (born at 32 weeks) and 36 weeks (born at 29 weeks) for preterm neonates, and had been diagnosed with retinoblastoma (seven patients) or low risk neuroblastoma (two patients), one stage 4s with massive hepatomegaly and respiratory compromise and one localised, unresectable without segmental chromosomal abnormalities presenting with spinal cord compression. Patient information is provided alongside details of individual patient treatment in Table 1. It should be noted that patient 001 was more than 4 weeks of age (52 weeks post-menstrual age) at the time of treatment and therefore falls outside the strict definition of a neonate. In all cases carboplatin was administered diluted in 5% dextrose as a 60 min intravenous infusion. Initial doses were based on body weight or body surface area, with therapeutic drug monitoring approaches used to achieve target carboplatin AUC values where required. Ethical approval was not required for this approach to treatment which is classed as the most appropriate clinical practice based on current evidence. Therapeutic drug monitoring in this setting was carried out at the request of the treating clinician.

### 2.2 Blood sampling and analysis

Blood samples (1 ml) for pharmacokinetic analysis were obtained from a central line before carboplatin infusion, 30 min after the start of infusion, at 60 min (end of infusion) and at 120–180 min after the start of infusion (60–120 min post-infusion). Plasma was separated from whole blood samples by centrifugation (1200*g*, 4 °C, 10 min), and 0.5 ml was then removed and placed in an Amicon Centrifree micropartition unit with a 30,000 MW cut-off (Millipore, Edinburgh, UK). This sample was centrifuged (1500*g*, 4 °C, 15 min) to obtain plasma ultrafiltrate for the determination of free carboplatin levels. Samples were sent by overnight courier, on dry ice and in an insulated container, to the Northern Institute for Cancer Research, Newcastle University. Platinum pharmacokinetic analyses were carried out by flameless atomic absorption spectrophotometry (AAS) using a Perkin–Elmer AAnalyst 600 graphite furnace spectrometer (Perkin-Elmer Ltd, Beaconsfield, UK). Free or unbound platinum levels were determined in plasma ultrafiltrates as described previously [19,20]. All samples were analysed in duplicate and values expressed as the average of these measurements. Duplicate values were within 15% of each other in all cases. Intra- and inter-assay coefficients of variation for a quality assurance sample had to be <10% for an assay to be valid. The limit of quantification (LOQ) for the AAS assay was 0.10 μg/ml.

### 2.3 Therapeutic drug monitoring

Carboplatin clearance and AUC were determined by Bayesian analysis following each dose of carboplatin using a two compartment model as described previously [20,21]. In brief, this allows the reliable estimation of carboplatin AUC through use of limited sampling approaches, with carboplatin clearance and AUC calculated based on data collected from previously studied paediatric patients aged 0–18 years. The two compartment model used was fitted to individual patient plasma ultrafiltrate concentrations determined by AAS using the Bayesian maximum a posterior (MAP) estimator and an error model derived from the previously defined population error model parameters. For patients being treated on a 3-day carboplatin schedule, dosing was adjusted on either day 2 or day 3, based on drug exposures and clearance values determined for day 1 and/or day 2, to achieve the desired target cumulative AUC. Carboplatin dose adjustments were recommended for all patients with day 1 (or days 1 and 2) AUC values >10% outside the target daily AUC. Dose adjustments were calculated based on the actual carboplatin clearance determined on day 1 (or days 1 and 2) and the remaining AUC to be achieved. Dosing on subsequent courses of carboplatin treatment was guided by the exposures observed on course 1, with therapeutic drug monitoring again carried out as appropriate.

### 2.4 Statistical analysis

Linear regression analysis and the Pearson correlation coefficient were used to indicate correlations between actual patient body weight and carboplatin clearance and between age and carboplatin clearance.

## 3 Results

### 3.1 Carboplatin treatment and dose adjustment

Patients received carboplatin either as single agent treatment (5/9 patients) or with additional chemotherapy including etoposide (4/9 patients) and vincristine (2/9 patients) as shown in Table 1. Real-time therapeutic drug monitoring approaches were used to either allow the modification of carboplatin dosage over 3 days of treatment to achieve a target cumulative AUC value, or to determine the AUC value achieved with the dosing regimen implemented, with a view to maintaining or altering the target AUC for course 2 of treatment based on the response and/or toxicity observed (as outlined in Table 1). Target cumulative carboplatin AUC values ranged from 5.2 to 7.8 mg/ml min over 3 days of treatment. Carboplatin doses were modified in real-time based on the daily AUC values observed in seven patients. In an additional patient, confirmation of the achievement of appropriate carboplatin AUC values, alongside observable clinical responses, allowed doses to be maintained on subsequent courses of treatment. The final patient had an AUC value calculated on day 1 of treatment, with a view to modifying carboplatin dosage on days 2/3, but died prior to treatment on day 2 due to complications relating to the size and position of the tumour (stage 4s neuroblastoma).

Initial doses of carboplatin administered on day 1 of course 1 of treatment ranged from 3.5–6.6 mg/kg/day, with day 1 AUC values of 0.8–2.7 mg/ml min achieved, reflecting a sixfold range in carboplatin clearance values of 3.4–19.4 ml/min. Cumulative AUC values calculated on courses of carboplatin chemotherapy where blood samples were collected were within 15% of the target value for the individual courses of treatment in all but one patient (12/13 courses of treatment), with dose modifications of up to 215% required to achieve target AUC values, based on the initial mg/kg dosing schedule implemented. The cumulative AUC achieved for patient 008 on course 2 of treatment was outside the desired ±15% AUC target window due to a shift in carboplatin clearance between days 1 and 3. Predicted AUC values that would have been achieved without the implementation of therapeutic drug monitoring were up to 100% outside of the target AUC (ranging from 2.4 to 9.7 mg/ml min), based on the carboplatin clearance values observed on each day of treatment. Table 2 shows the carboplatin doses administered and AUC values achieved in each of the seven patients where doses were modified in real-time over 3 day treatment periods, based on the daily AUC values observed.

**Fig. 1:**
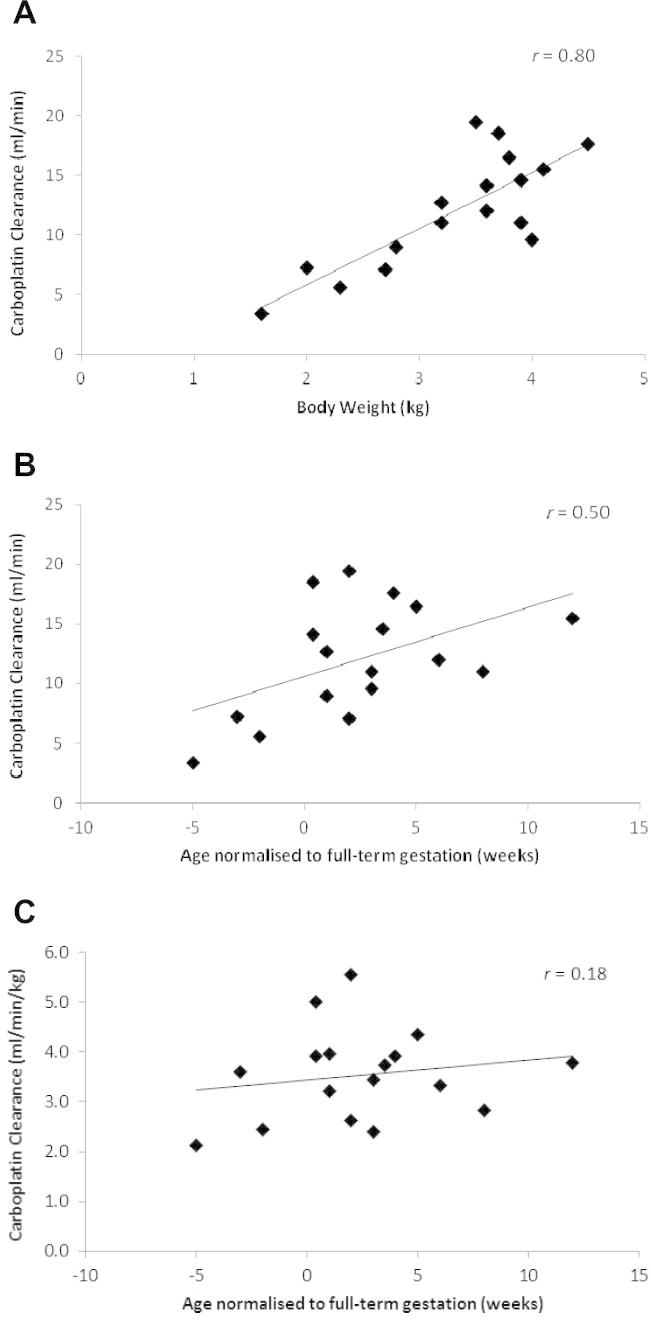
Relationship between patient body weight and carboplatin clearance (A), between patient age (normalised to full-term gestation for preterm neonates) and carboplatin clearance (B) and between patient age (normalised to full-term gestation for preterm neonates) and carboplatin clearance (normalised to body weight) (C).

**Fig. 2:**
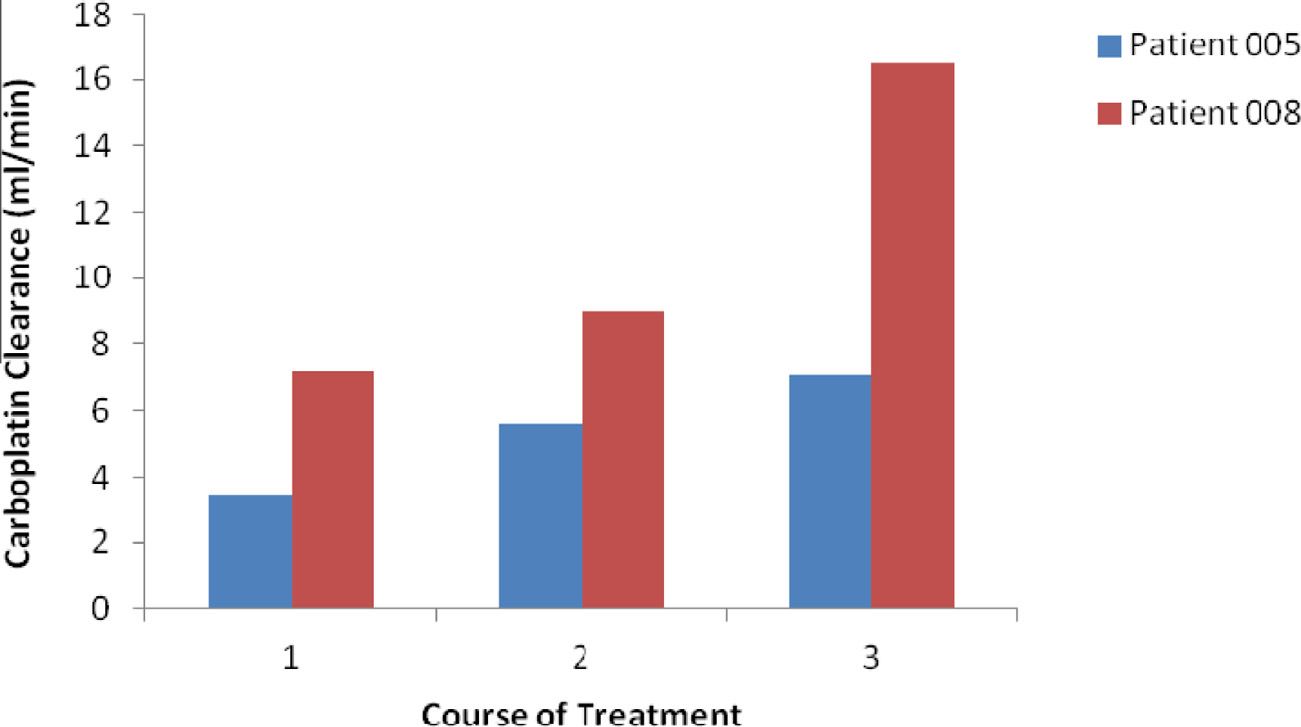
Change in carboplatin clearance values measured across three courses of treatment in two patients.

Table 3 provides a summary of carboplatin doses administered and carboplatin clearance values observed on course 1 (nine patients), courses 1 and 2 (six patients) and courses 1–3 (two patients). The relationship between patient body weight and carboplatin clearance for all courses of chemotherapy where samples for pharmacokinetic analysis were obtained (i.e. 17 courses across nine patients), is shown in Fig. 1A. Similarly, relationships between patient age (normalised to full-term gestation for neonates born preterm) and carboplatin clearance, and between patient age (normalised to full-term gestation for neonates born preterm) and carboplatin clearance normalised to body weight are shown in Fig. 1B and C, respectively. Carboplatin clearance and AUC values were determined across three consecutive courses of chemotherapy in two patients, with mean clearance values increasing from 3.4 to 7.1 ml/min in patient 005 and from 7.2 to 16.5 ml/min in patient 008, representing increases of 210% and 230%, respectively (Fig. 2), over 7–8 weeks of treatment.

### 3.2 Treatment response and toxicity

Of the seven patients treated for retinoblastoma, complete remission was observed in all cases following 2–6 courses of single agent carboplatin or in combination with the additional anticancer drugs and treatment approaches outlined in Table 1. All of these patients remain in remission with follow-up periods currently ranging from 5 months to >5 years. One patient relapsed on two separate occasions but now remains in remission following additional treatment with carboplatin, etoposide and vincristine alongside local laser treatment. Grade 3/4 haematological toxicities including neutropenia and anaemia were observed in 4/7 patients with retinoblastoma, with additional toxicities including diarrhoea, vomiting and a septic episode. Only one patient experienced ototoxicity in the form of sensorineuronal deafness and in this case it was unclear to what extent this was attributable to carboplatin treatment as the patient had pre-existing hearing loss and associated 13q deletion syndrome. No patients studied experienced renal toxicity.

One patient with low risk, localised, unresectable neuroblastoma experienced a complete response after two courses of carboplatin treatment and subsequent spontaneous tumour regression. No significant toxicity including renal or hearing impairment was observed and the patient remains disease free with 5 year follow-up. The second patient with low risk, stage 4s neuroblastoma died prior to carboplatin treatment on day 2 of the first course of treatment due to complications relating to tumour burden.

## 4 Discussion

The limited knowledge of drug disposition in preterm and full-term neonates for the vast majority of anticancer drugs means that defining appropriate dosing regimens for this patient population is a challenging task. The current study provides real world data which for the first time facilitates the provision of scientifically-based dosing guidelines for the treatment of preterm and full-term neonates with carboplatin. The incidence of ototoxicity in children treated with carboplatin has previously been highlighted as a major concern, with incidence rates of carboplatin-induced ototoxicity ranging from 8 to 50% across various tumour types [18,22–24]. In this respect, a recently published study by Qaddoumi et al. highlighted the fact that younger children with retinoblastoma are at an increased risk of experiencing ototoxicity, with children <6 months of age 21-times more likely to have sustained hearing loss than older children following carboplatin treatment [18]. Indeed, in this study 9/10 of patients who experienced sustained hearing loss were between 0.4 and 4.5 months of age at the time of treatment, as compared to an age range of 0.5 months to 13.6 years in 50 patients who experienced no hearing loss; with median ages of 2.3 versus 10.3 months in these two groups respectively [18].

The data presented in the current study highlight the benefits of utilising therapeutic drug monitoring to target carboplatin AUC values, with achieved AUC values within 15% of the target on 12/13 courses where this approach was instigated. This allowed for the achievement of cumulative AUC values of 5.0–8.2 mg/ml min, based on a target AUC range of 5.2–7.8 mg/ml min, as opposed to a range of 2.9–9.7 mg/ml min which would have been achieved if no therapeutic drug monitoring had been implemented. In addition to the possibility that reduced carboplatin dosing implemented in neonates based on therapeutic drug monitoring may have resulted in the avoidance of toxicity in some of these patients, it is also important to consider the potential clinical impact of dose increases in those patients with lower drug exposures prior to drug monitoring. Marked carboplatin dose increases in these patients, as observed most notably in this study in the case of patients 002 and 008, not only avoid potential gross under-treatment, but may also help to negate an inaccurate judgement of chemo-insensitivity and resultant change to second line treatments which may be less effective and more toxic.

The range of initial carboplatin dosing approaches taken by different UK clinical centres on day 1 of treatment for this patient population highlights the uncertainties that exist and the lack of guidance available to treating clinicians. While in older children doses of carboplatin are frequently based on GFR, this is not possible within the first few days and weeks of life, predominantly due to the fact that radioactive and contrast media markers utilised for GFR measurement are not recommended for use in neonates by most clinicians. In addition, the collection of 24 h urine samples is not feasible in neonates and the interpretation of results can be difficult. Initial doses were therefore predominantly based on BW-based or BSA-based dosing, with doses ranging from 3.5 to 6.6 mg/kg/day or 100 to 200 mg/m^2^. While therapeutic monitoring over 3 days of treatment largely negates the differences in initial dosing regimens in the patients included in the current study, it is important to analyse the data generated appropriately in order to provide rational dosing guidelines to be utilised when therapeutic drug monitoring approaches are not possible.

**Fig. 3:**
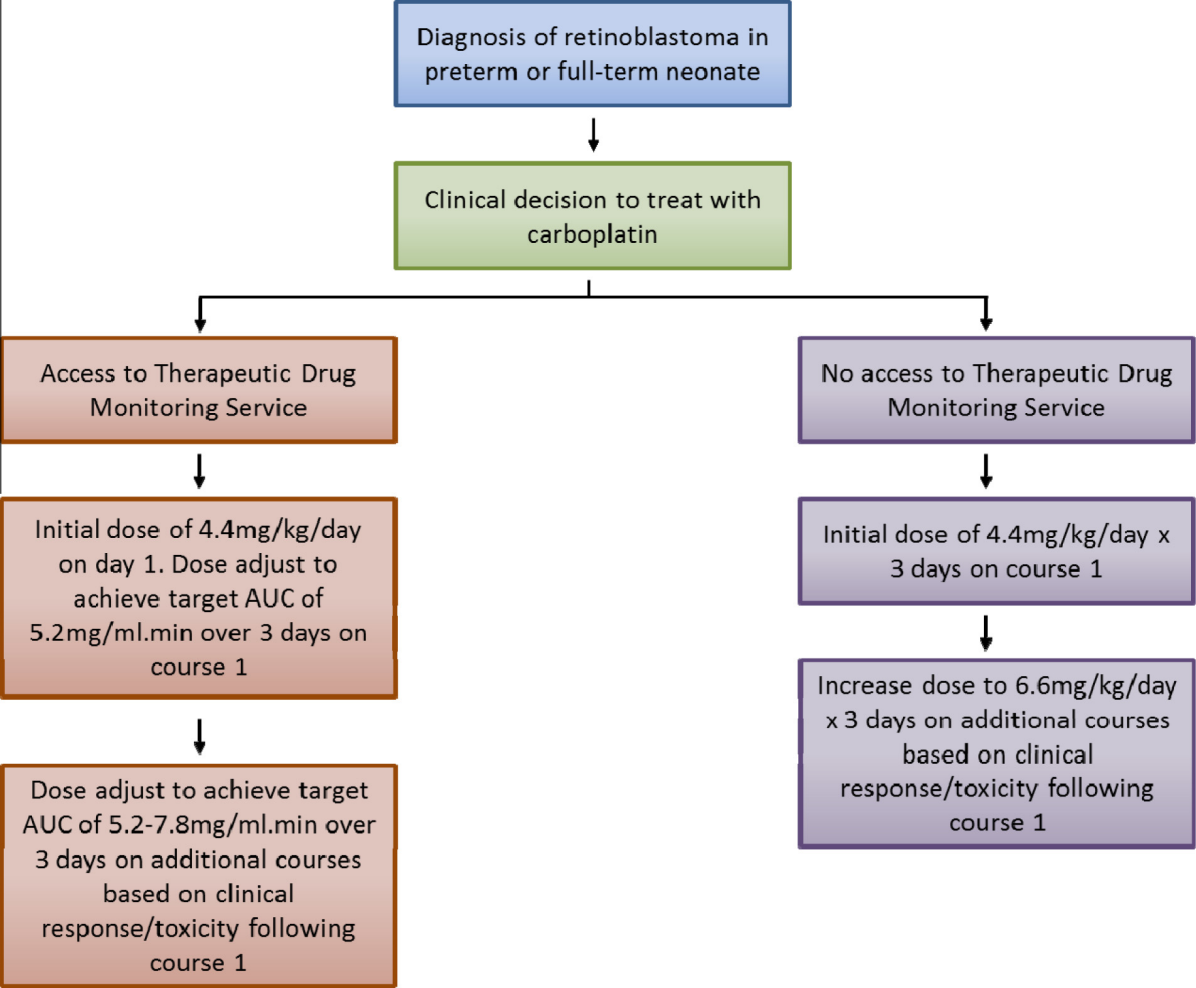
Carboplatin dosing guidance flow diagram for the treatment of retinoblastoma in preterm and full-term neonates up to 3 weeks of age.

Based on the relatively good correlation observed between patient BW and carboplatin clearance, BW-based dosing would appear to be the most pertinent approach to recommend in the absence of therapeutic drug monitoring. This is supported by previously published studies which have investigated the impact of various factors on renal function maturation in neonates and identified weight as the best standard for prediction of GFR [25,26]. In addition, data from several clinical trials have raised concerns over the use of BSA-based dosing as compared to BW-based dosing in infants with retinoblastoma [18,27,28]. In terms of an appropriate dose for treatment, we have generally utilised an initial target AUC value of 5.2 mg/ml min for the first course of treatment. Clinicians have then either opted to maintain this target AUC on additional courses of treatment, or have opted to increase the dose to target an AUC of 7.8 mg/ml min, depending on the response and level of toxicity observed. This will of course depend on whether or not carboplatin is being used alone or whether there are other chemotherapeutics being used in combination which need to be considered. Based on a median AUC of 1.325 mg/ml min being equivalent to a dose of 3.3 mg/kg, as used to develop the original carboplatin paediatric dosing formula by Newell et al. [12], we would recommend an initial starting dose of 4.4 mg/kg/day for 3 days of treatment on course 1, equivalent to a target AUC of 5.2 mg/ml min. This can be increased to a dose of 6.6 mg/kg/day for 3 days of treatment on course 2 if this was felt likely to be beneficial, based on the observed response and toxicity following course 1. Similarly, these guidelines do not preclude further dose increases above 6.6 mg/kg/day being employed where lack of response and toxicity is observed at this dose. In the current study the equivalent mg/kg/day dosing regimens required to obtain target AUC values of 5.2 and 7.8 mg/ml min were 3.2–7.2 mg/kg/day and 5.5–13 mg/kg/day respectively, therefore clearly some patients required higher doses for the achievement of these target exposures. Fig. 3 provides a carboplatin dosing guidance flow diagram for the treatment of preterm and full-term neonates with retinoblastoma, based on the data generated in the current study.

When considering the results obtained in the current study, it is important to consider children with neuroblastoma and retinoblastoma in the neonatal period as distinct populations, each with its own unique issues. In the neuroblastoma population, there is a potentially life-threatening condition that has a risk of causing tumour lysis syndrome and acute changes in GFR during induction chemotherapy, but where response to treatment and long-term outcome after initial treatment are very good. As long-term chemotherapy treatment is unlikely to be needed, the aim of treatment in this scenario is to save the life of the neonate, while avoiding life-threatening complications with limited cycles of treatment. The population with retinoblastoma has the potential for life-threatening illness, but the major issue is preservation of sight without compromising the excellent chance of cure or causing co-morbidities. In this situation treatment is likely to be prolonged and may need to be repeated, with the aim of administering an appropriate amount of chemotherapy to control the disease, while limiting potentially long-term side-effects such as ototoxicity.

In summary, we would recommend that carboplatin therapeutic drug monitoring approaches are utilised in preterm and full-term neonates whenever feasible, particularly bearing in mind the marked increases in carboplatin clearance observed over several courses of treatment. While allometric scaling with BW to an exponent of 0.75 or BSA represent appropriate approaches for the achievement of comparable exposures across a wide age range for many drugs, BSA-based dosing would not be appropriate for carboplatin due to its reliance on renal clearance and the disproportionally low GFR observed in infants. As observed carboplatin AUC values are highly variable even with BW-based dosing, therapeutic drug monitoring should be recommended. Such approaches may be particularly relevant in the case of neonates with retinoblastoma, where treatment within the first few weeks or months of life may lead to an increased risk of visual defects following treatment [29,30]. In this clinical scenario it is clearly important to reduce the possibility of sensorineural hearing loss, which is likely to have an even greater impact on the quality of life of a child who also has visual impairment. However, we appreciate that therapeutic drug monitoring approaches will not be possible in many cases. The guidance provided in relation to the use of BW-based dosing to target the equivalent of AUC values of 5.2–7.8 mg/ml min reflects the most appropriate approach to dosing based on currently available data. It is essential that while continuing to ensure positive clinical outcomes in patients being treated with cytotoxic anticancer drugs such as carboplatin, we also strive to minimise the side-effects of treatment. The current study uses evidence from pre-term and full-term neonates treated with carboplatin in the first few weeks of life to provide dosing guidance which will positively impact on the treatment of future patients.

## Role of the funding source

This work was supported in part by Cancer Research UK and the Experimental Cancer Medicine Centre Network. Neither funding body played a role in the study design, the collection, analysis or interpretation of data, the writing of the report or the decision to submit the article for publication.

## Conflict of interest statement

None declared.

## Figures and Tables

**Table 1:** Patient characteristics and treatment details.

Patient	Tumour type	Age at diagnosis	Initial treatment and carboplatin dosing regimen	Number of courses of carboplatin	TDM approach taken
001	Retinoblastoma (bilateral)	52 weeks gestational age (born at 28 weeks)	Single agent carboplatin 100 mg/m^2^/day ×3; target AUC 5.2 mg/ml min^a^^,^^d^	6	TDM used to achieve target AUC on course 1. Good response and tolerability so equivalent mg/kg dosing used for courses 2–6
002	Retinoblastoma (bilateral)	3 days (gestational age of 40 weeks)	Single agent carboplatin to target AUC 5.8 mg/ml min^a^	6	TDM used to achieve target AUCs on courses 1/2. Good response after courses 1 and 2 so equivalent mg/m^2^ dosing used for courses 3–6
003	Neuroblastoma (low risk stage 4s)	2 weeks (gestational age of 37 weeks)	Carboplatin (6.6 mg/kg)/etoposide (5 mg/kg)	1	TDM proposed to determine AUC achieved on course 1
004	Neuroblastoma (low risk, localised, un-resectable)	3 days (gestational age of 40 weeks)	Carboplatin (4.4 mg/kg)/etoposide (5 mg/kg)	2	TDM used to determine AUCs achieved on courses 1 and 2. Good response and tolerability following mg/kg dosing on both courses
005	Retinoblastoma (bilateral)	35 weeks gestational age (born at 32 weeks)	Single agent carboplatin (6.6 mg/kg); target AUC 5.2 mg/ml min^a^	5	TDM used to achieve target AUC on courses 1–3. Good response and tolerability so equivalent mg/kg dosing used for courses 4/5
006	Retinoblastoma (bilateral)	3 weeks (gestational age of 40 weeks)	JOE^b^ ×6 cycles; carboplatin dose 100 mg/m^2^/day ×3; target AUC 7.7 mg/ml min	4	TDM used to achieve target AUC on course 1. Good response and tolerability so equivalent mg/m^2^ dosing used for courses 2–4
007	Retinoblastoma (left)	1 week (gestational age of 40 weeks)	JOE^b^ ×3 cycles; carboplatin dose 6.6 mg/kg/day ×3; target AUC 7.8 mg/ml min^c^	3	TDM used to achieve target AUCs on courses 1/2. Good response and tolerability so equivalent mg/kg dosing used for course 3
008	Retinoblastoma (bilateral)	36 weeks gestational age (born at 29 weeks)	Single agent carboplatin to target AUC 5.2 mg/ml min a	3	TDM used to achieve target AUC on courses 1–3
009	Retinoblastoma (right)	3 weeks (gestational age of 40 weeks)	Single agent carboplatin (6.6 mg/kg) to target AUC 5.8 mg/ml min a	2	TDM used to achieve target AUC on courses 1 and 2

TDM – therapeutic drug monitoring.

a Patient also received local laser treatment.

b JOE – vincristine, etoposide and carboplatin.

c Patient also received thermotherapy.

d Patient also received cryotherapy.

**Table 2:** Carboplatin doses administered and AUC values achieved on courses of treatment where therapeutic drug monitoring was used to adjust dosing as compared to BW-based dosing approaches.

Patient	Treatment course	Target AUC (mg/ml min)	Carboplatin dose (mg)		AUC (mg/ml min)	
			Actual (TDM)	BW-based^a^	Actual (TDM)	BW-based^b^
001	1	5.2	80	78	5.2	5.1

002	1	5.8	105	72	5.8	3.9
2	7.8	136	120	7.8	6.8

005	1	5.2	15.5	33	5.0	9.7
2	7.8	38	36	7.1	6.4
3	7.8	55	44	7.7	6.2

006	1	7.7	71	75	7.3	7.8

007	1	7.8	80	60	6.4	4.8

008	1	5.2	43	21	6.1	2.9
2	7.8	70	29	7.8	3.2
3	7.8	129	39	7.8	2.4

009	1	5.8	63	63	5.8	5.8
2	5.8	90	78	8.2	7.1

TDM – therapeutic drug monitoring; BW – body weight.

a BW-based dosing regimens varied from 3.5 to 6.6 mg/kg/day depending on treatment centre and protocol used to guide initial treatment.

b BW-based AUC values are predicted from the carboplatin clearance values determined during treatment, reflecting the values that would have been observed if BW-based dosing had been carried out.

**Table 3:** Carboplatin clearance values observed on courses 1–3 of treatment.

Patient	Carboplatin course 1				Carboplatin course 2				Carboplatin course 3			
	BW (kg)	SA (m^2^)	Daily dose (mg)	Cl^a^ (ml/min)	BW (kg)	SA (m^2^)	Daily Dose (mg)	Cl^a^ (ml/min)	BW (kg)	SA (m^2^)	Daily dose (mg)	Cl^a^ (ml/min)
001	4.1	0.26	26	15.5								
002	3.7	0.25	24–41	18.5	4.5		40	17.6				
003	3.5	0.24	15	19.4								
004	3.6		15	14.1	3.9	0.26	17	14.6				
005	1.6		4.5–11	3.4	2.3		11–16	5.6	2.7		15–20	7.1
006	4.0	0.26	23–25	9.6								
007	3.2		20–30	12.7	3.6		30	12.0				
008	2.0	0.16	7–18	7.2	2.8	0.20	18	9.0	3.8	0.20	25	16.5
009	3.2	0.21	21	11.0	3.9	0.26	26–38	11.0				

BW – body weight; SA – surface area; Cl – clearance.

a Mean clearance values shown where carboplatin pharmacokinetics determined on more than one day of a particular course of treatment.
